# Exploring the novel duo of Reticulocalbin, and Sideroflexin as future biomarker candidates for Exacerbated Chronic Obstructive Pulmonary Disease

**DOI:** 10.1186/s12014-024-09459-8

**Published:** 2024-02-14

**Authors:** Sonu Das, Supriya Adiody, Jinsu Varghese, M Vanditha, Evelyn Maria, Mathew John

**Affiliations:** 1 Biochemistry and Phytochemistry Research Division, Jubilee Centre for Medical Research, Jubilee Mission Medical College and Research Institute, Thrissur, Kerala India; 2 Department of Zoology, St. Thomas College, Kozhencherry, Affiliated to Mahatma Gandhi University, Kerala India; 3 Department of Pulmonary Medicine, Jubilee Mission Medical College and Research Institute, Thrissur, Kerala India; 4 Department of Biochemistry, Amrita Institute of Medical Sciences, Kochi, Kerala India

**Keywords:** Fe-S biogenesis, Unfolded protein response, ECM remodelling, Sideroflexin-4, Liprinα-3

## Abstract

**Background:**

COPD is a complex respiratory disorder with high morbidity and mortality rates. Even with the current conventional diagnostic methods, including circulating inflammatory biomarkers, underdiagnosis rates in COPD remain as high as 70%. Our study was a comparative cross-sectional study that aimed to address the diagnostic challenges by identifying future biomarker candidates in COPD variants.

**Methods:**

This study used a label-free plasma proteomics approach that combined mass spectrometric data with bioinformatics to shed light on the functional roles of differentially expressed proteins in the COPD lung microenvironment. The predictive capacity of the screened proteins was assessed using Receiver Operating Characteristic (ROC) curves, with Western blot analysis validating protein expression patterns in an independent cohort.

**Results:**

Our study identified three DEPs—reticulocalbin-1, sideroflexin-4, and liprinα-3 that consistently exhibited altered expression in COPD exacerbation. ROC analysis indicated strong predictive potential, with AUC values of 0.908, 0.715, and 0.856 for RCN1, SFXN4, and LIPα-3, respectively. Validation through Western blot analysis confirmed their expression patterns in an independent validation cohort.

**Conclusions:**

Our study discovered a novel duo of proteins reticulocalbin-1, and sideroflexin-4 that showed potential as valuable future biomarkers for the diagnosis and clinical management of COPD exacerbations.

**Supplementary Information:**

The online version contains supplementary material available at 10.1186/s12014-024-09459-8.

## Introduction

Chronic Obstructive Pulmonary Disease (COPD), a multifaceted respiratory disorder, is characterized by chronic inflammation within the airways and lung tissues, caused by a complex interplay between the immune response and environmental factors. There is significant concern over the global impact of COPD, with 384 million people affected in 2020 alone. Despite the high morbidity and mortality rate in COPD, 70% of the disease remains underdiagnosed, contributing to 3.23 million deaths per year, accounting for 5.7% of all global fatalities [1, 2].

The Global Initiative for Chronic Obstructive Lung Disease (GOLD) classifies COPD into two categories: the milder stable form of COPD interspersed with episodes of worsening symptoms defined as exacerbation COPD. At present, the gold standard for COPD diagnosis is considered to be spirometry, supported by biochemical and hematological tests for generalized markers of inflammation, imaging and physical examination. However, these methods have their own limitations, being inconclusive and time-consuming [3]. A significant limitation of the existing approaches is the lack of precision in COPD diagnosis and characterization, leading to high rates of underdiagnosis [4]. Moreover, these conventional diagnostic methods could not distinguish between stable and exacerbation COPD.

With the emergence of proteomics, several studies attempting to identify circulating inflammatory proteins as diagnostic markers were carried out for the early detection and therapeutic management of COPD. However, proteomics studies to date have failed to identify a specific biomarker for COPD other than generalized markers such as haptoglobin, transferrin, fibrinogen and orosomucoid, which are glycoprotein-derived mediators of inflammation [5]. These proteins could only implicate general inflammation in the body and do not warrant diagnosis of lung-specific chronic inflammation as in COPD, nor could they distinguish between stable and exacerbated COPD. This major limitation in proteomic studies in the past could be attributed to lower sample size, retrospective study design and the use of a labelled approach targeting acute phase reactants.

Our study sought to bridge this gap in COPD diagnosis by using a label-free plasma proteomic approach, wherein we unmasked a novel duo of proteins that could effectively delineate exacerbation COPD from the stable variant. By adopting a label-free approach, we were able to obtain higher proteome coverage, facilitating the identification of specific proteins with noteworthy functionality in COPD exacerbation. The DEPs we identified were not acute phase reactants or glycoproteins that implicate generalized inflammation in the system. These DEPs showed functional connectivity to inflammatory signaling in the lung microenvironment and a mechanistic association with pulmonary function in COPD exacerbation. Our study is a pioneering work to identify specific inflammatory proteins in COPD exacerbations that show great promise as future potential biomarkers revolutionizing the diagnosis, delineation and therapeutic management of exacerbation COPD.

## Materials and methods

### Sample selection

Our study was a comparative cross-sectional plasma proteome profiling study conducted in a tertiary care hospital in accordance with the Institutional Ethics Committee and the Helsinki Declaration of 1975, as revised in 2000 (IEC study ref no: 45/20/IEC/JMMC&RI). All study group participants ranged in age from 40 to 80, and blood samples were collected after written informed consent was obtained.

The groups for the proposed study were classified as follows:

- Group A: Healthy controls (HC).
- Group B: Stable COPD (SC).
- Group C: Exacerbated COPD (EC).

The study was a prospective study carried out in a total of 36 plasma samples (12 HC, 12 SC and 12 EC) collected prior to treatment initiation. COPD patients with autoimmune diseases, asthma overlap, lung cancer, COVID-19, tuberculosis and other chronic inflammatory conditions were excluded from the study.

Stable and exacerbated COPD patients were diagnosed by a pulmonologist on the basis of clinical presentation and spirometry values. The samples in the discovery phase were age-matched and sex matched with respect to control and screened based on their strict adherence to GOLD classification criteria as well as on the homogeneity of their baseline biochemical parameters. Grouping and sub-grouping of the COPD variants were done based on CAT and mMRC questionnaires along with assessment of other biochemical markers such as CRP. Detailed demographic data for the samples in the discovery cohort are given in Supplementary Table 1. A representative chest X-ray of COPD in comparison to healthy control indicating hyperinflammation is given in Supplementary figure S1 (representatives of the discovery cohort).

### Sample collection

Blood was collected in EDTA vacutainers from the participants in the age group between 40 and 80 years. The samples were centrifuged at 3000 rpm for 10 min to separate the plasma. The separated plasma samples were stored at -80 °C until further analysis.

### Depletion of abundant plasma proteins

Plasma samples were subjected to depletion, wherein fourteen interfering high-abundance proteins, namely albumin, IgG, antitrypsin, IgA, transferrin, haptoglobin, fibrinogen, alpha2-macroglobulin, alpha1-acid glycoprotein, IgM, apolipoprotein AI, apolipoprotein AII, complement C3 and transthyretin were removed from plasma samples to facilitate the unmasking of lower abundant proteins using Multiple Affinity Removal Spin Cartridge Human 14 (MARS Hu-14) [Cat. No: 5188–6560]. The flowthrough, eluent and crude plasma samples were run through SDS‒PAGE to validate the removal of the 14 proteins in the flowthrough fraction, as shown in supplementary figure S2.

### Protein concentration

The flow-through fraction from each sample was passed through 5 KDa Molecular Weight Cut-Off Spin Concentrators [Cat. No: 5185–5991] for salt depletion and buffer exchange. The protein samples were further concentrated by freeze drying using an Operon FDU 7003 speed vac lyophilizer. Protein quantitation of the samples was performed using the Thermo ScientificPierce BCA Protein Assay Kit [Cat. No: 23,225]. The protein concentration of all the samples for proteomic analysis was normalized to 1 mg/mL in 50 mM ammonium bicarbonate (ABC) (pH 7.8).

### Tryptic digestion

Tryptic digestion was carried out using standard protocols. Digestion buffer used in this study was 50 mM ammonium bicarbonate (ABC) (pH 7.8). Approximately 100 µg of protein from each sample were subjected to in-solution tryptic digestion. The samples were first reduced using 100 mM 1,4-dithiothreitol (DTT) in ammonium bicarbonate (initial concentration) [Sigma Aldrich, CAS No: 3483-12-3] for 30 min at 60 °C. Alkylation of the samples were done by incubating them in 200 mM iodoacetamide in ABC (initial concentration) [Sigma Aldrich, CAS No: 144-48-9] in the dark at room temperature for 30 min. The samples were digested using MS-grade trypsin in ABC [Sigma Aldrich, Cat no: T6567] in a 1:25 ratio of trypsin to protein, incubated for 17 h at room temperature and enzymatic reaction was stopped using 1.0% formic acid, incubated for 20 min at room temperature. The digested peptide solution was centrifuged at 14,000 rpm for 12 min and the supernatant collected.

### LC‒MS /MS

The proteomic profiling was conducted using liquid chromatography tandem mass spectrometry [LC–MS/MS] at the DBT-SAHAJ National Facility for Mass Spectrometry at Rajiv Gandhi Centre for Biotechnology in Thiruvananthapuram, Kerala. Relative protein quantification was performed using ESI-nano LC‒MS/MS (nanoACQUITY UPLC® chromatographic system by Waters in Manchester, UK).

The peptide sample was injected in partial loop mode in 5 µl loop (final injection volume was 1 µl which contains around 1 micrograms of tryptic digested peptides). Each sample was injected in duplicate, with two injections performed per sample. Water was used as solvent A and acetonitrile was used as solvent B. All solvents for the UPLC system contained 0.1% formic acid. The tryptic peptides were trapped and desalted on a trap column (Symmetry® 180 μm * 20 mm C18 5 μm, Waters) for 1 min at a flow rate of 15 µl/min. The trap column was placed in line with the reversed-phase analytical column, a 75 μm i.d. * 200 mm HSS T3 C18 (Waters) with particle size of 1.8 μm. Peptides were eluted from the analytical column with a linear gradient of 1 to 40% solvent B over 55.5 min at a flow rate of 300 nl/min followed by a 7.5 min rinse of 80% solvent B. The backpressure varied between 450 and 650 bar. The temperature of the column oven was 35 °C. Chromatography was performed in reversed-phase mode, and the autosampler temperature was set at 4 °C. The *lock mass*, leucine enkephalin (Sigma) (positive ion mode [M + H] + = 556.2771) for mass correction was delivered from the auxiliary pump of the UPLC system through the reference sprayer of the NanoLockSpray™ source at a flow rate of 500 nl/min. Each sample was injected in duplicate with blank injections between each sample. (See Supplementary Table 2).

### Mass spectrometric conditions

The MS runs were performed using data independent mode (MSE) coupled with ion mobility-enabled separation. The study has used ion mobility for this label free proteomic approach as it provides an extra layer of separation, relying on the molecular size and shape as its determining factors. The advantages of ion mobility is better isomer separation, noise elimination, cleaner spectra, and better compound identification. Mass spectral analysis of eluting peptides from the nanoACQUITY UPLC® was carried out on a SYNAPT® G2 High-Definition MS™ System (HDMS^E^ System, Waters). It is a hybrid, quadrupole, ion mobility, orthogonal acceleration, time-of-flight mass spectrometer controlled by MassLynx4.1 SCN781 (Waters Corporation, Milford, MA, USA) software. The instrument settings were: nano-ESI capillary voltage – 3.4 KV, sample cone − 40 V, extraction cone − 4 V, IMS gas (N_2_) flow − 90 (ml/min). To perform the mobility separation, the IMS T-Wave™ pulse height is set to 40 V during transmission and the IMS T-Wave™ velocity was set to 800 m/s. The travelling wave height was ramped linearly over 100% of the IMS cycle between 8 and 20 V.

All analyses were performed using positive mode ESI using a NanoLockSpray™ source. The lock mass channel was sampled every 45 s. The time-of-flight analyzer (TOF) of the mass spectrometer was calibrated with a solution of sodium iodide at a concentration of 2 µg/µl (micrograms per microlitre). This calibration set the analyzer to detect ions in the range of 50–2000 *m/z*. The data acquisition was done in *continuum* format. The data was acquired by rapidly alternating between two functions – Function-1 (low energy) and Function-2 (high energy). In Function-1, we acquire only low energy mass spectra (MS) and in Function-2, we acquire mass spectra at elevated collision energy with ion mobility (HDMS^E^). In Function-2, collision energy was set to 4 eV in the Trap region of mass spectrometer and is ramped from 20 eV to 45 eV in the Transfer region of mass spectrometer to attain fragmentation in the HDMS^E^ mode. The *continuum* spectral acquisition time in each function was 0.9 s with an interscan delay of 0.024 s.

### Data analysis conditions

The data analysis software was Progenesis QI for Proteomics V4.2 software (Non-Linear Dynamics, Waters). Organism for protein identification: “*Homo sapiens*”- reviewed protein entries only, Database source: UniProt database (https://www.uniprot.org/). False positive rate: 1, missed cleavage: 1, Number of fragments per peptide: 1, Number of fragments per protein: 3, Number of peptides per protein: 1, Variable modification: methionine oxidation, fixed modification: cysteine carbamidomethylation. The total number of peptides ≥ 2, number of unique peptides ≥ 1, max fold change ≥ 1.5, corrected p-value using an FDR approach < 0.05. Deconvolution of the ESI- mass spectrometric data was carried out to normalize and quantify the detected peptides, by applying a ratiometric data in log space along with a mean absolute deviation outlier filtering, done using the ‘normalize to all proteins’ option in in Progenesis QI.

### Western blot

Proteins with interesting functionality in the context of COPD were validated using Western blot in an independent sample set. Baseline characteristics of the samples in validation cohort is given in supplementary Table 3. This study validated two proteins namely, reticulocalbin-1, and sideroflexin-4, in an independent cohort of 24 samples (12 HC and 12 EC). These proteins were selected for validation in exacerbation COPD based on their consistent differential expression with respect to healthy control groups and the milder stable COPD. Liprinα-3 another differentially expressed protein showed a consistent downregulation in COPD with respect to healthy controls. Plasma samples for western blotting were processed as follows. The samples were subjected to depletion of abundant proteins using a Multiple Affinity Removal Spin Cartridge Human 14 (MARS Hu-14) [Cat. No: 5188–6560], protein concentrated and normalized to 1 mg/mL. The proteins were separated using 10% polyacrylamide SDS- PAGE. Gels were transferred onto 0.45 µM PVDF membrane [Millipore Immobilon -FL PVDF membrane, [Cat.No: 05317] using the Bio-Rad Trans-Blot Turbo Transfer system. Blocking was performed using 5% BSA [Cas No:

9048-46-8] in TBS-T. Membranes were probed with primary antibodies in blocking buffer overnight at 4 °C. Membranes were washed in TBS-T thrice and TBS twice and subsequently incubated in secondary antibody in blocking buffer for one hour. The primary antibodies were Reticulocalbin-1 polyclonal antibody [Invitrogen-PA5 87,992], Sideroflexin-4 polyclonal antibody [Invitrogen-PA5 98,509] and Liprin α-3 antibody [Abcam-Ab-180,888]. The blots for the three proteins were visualized using Clarity Western ECL substrate [Cat.No: 1,705,060] in the GBox Chemidoc system.

### Bioinformatics analysis

Three groups were defined for the comparison of differentially expressed proteins delineating stable and exacerbated COPD from each other and with respect to healthy controls.

- They were: Group C Vs A (EC Vs HC) – DEPs in exacerbated COPD with respect to control.
- Group B Vs A (SC Vs HC) – DEPs in stable COPD with respect to control.
- Group B Vs C (SC Vs EC) – DEPs in stable COPD with respect to exacerbated COPD.

For protein identification, only unique peptides with a high confidence score were used. A Venn diagram was created using Venny (bioinfogp.cnb.csic.es/tools/venny/index.html) to identify the proteins unique to and common to the COPD variants with respect to each other and to healthy controls.

The uniformity of the samples in the two groups was determined through the application of a multivariate statistical technique, partial least squares discriminant analysis (PLS-DA) using Metaboanalyst, Ver.5.0 (www.metaboanalyst.ca). PLS-DA was used to study the intergroup and intragroup similarities and differences among protein expression from different samples, wherein the abundance of proteins identified in each technical replicate were used independently and missing values were replaced by zeroes. Subsequently, Metaboanalyst was used to generate volcano plots depicting the differential expression of proteins between the stable COPD, exacerbated COPD, and healthy control groups. The cut off threshold values for the volcano plot were set with adjusted *p value* ≤ 0.05 and log_2_FC in the range of -1.5 to 1.5.

A heatmap was constructed to visualize the hierarchical clustering of the top 50 DEPs common to stable and exacerbated COPD with respect to healthy controls, with clustering of proteins based on PLS-DA, normalized data source and Euclidean distance.

The differentially expressed proteins (DEPs) in stable and exacerbated COPD were mapped and functionally annotated for their biological processes (BP) using the Databases for Annotation, Visualization and Integrated Discovery (DAVID) against the Human proteome (https://david.ncifcrf.gov/) and the pathway enrichment analysis of these DEPs were carried out using KOBAS (http://kobas.cbi.pku.edu.cn). The biological processes (BP) with a p value ≤ 0.05 corrected using Benjamini Hochberg method were considered significantly enriched and visualized in Metascape (https://metascape.org).

The predictive potential of the selected DEPs, reticulocalbin-1, sideroflexin 4 and liprin α-3 in COPD exacerbation were analyzed using classical univariate receiver operator characteristic curve (ROC) with 95% confidence interval setting and their AUC values were assessed in Metaboanalyst Ver.5.0.

### Statistical analysis

Statistical analysis and visualization were performed in SPSS version 25. The Mann‒Whitney U test was used for between-group comparisons in western blot band quantitation, and the results were represented as mean ± percentage deviation in a bar plot.

For filtering proteins for downstream analysis, proteins with two or more peptide counts and at least one unique peptide were selected. Proteins with a differential fold change greater than or equal to 1.5 were selected, with a corrected p-value less than 0.05, that were obtained using an optimised FDR approach. The FDR approach was optimised by producing a list of q-values based on p-value distribution characteristics which helped to establish how many of the significant values were actually false positives. Independent t-tests were performed for the relative quantitation between healthy controls and COPD variants for all the bioinformatics analysis.

## Results

**Fig. 1:**
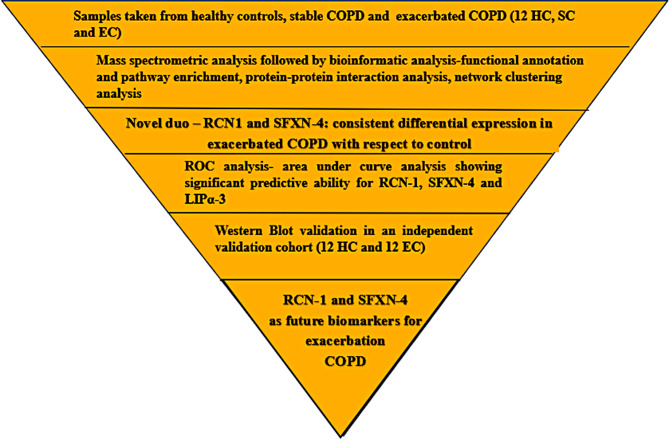
Schematic representation of the plasma proteomics workflow: A step-by step downstream analysis of plasma proteome in COPD subvariants with respect to healthy control

The present study adopted a label-free plasma proteomics approach to study COPD variants in comparison to healthy controls. A schematic representation of the proteomics workflow is given in Fig. 1. A total of 36 plasma samples (12 SC, 12 EC, 12 HC) were analysed by high-definition mass spectrometry in the discovery phase.

A total of 250 proteins were identified to be differentially expressed in stable and exacerbated COPD with respect to healthy controls. After application of FDR 1% and screening based on max fold change set at 1.5; 68 differentially expressed proteins in stable COPD and 65 differentially expressed proteins in exacerbated COPD were identified with respect to healthy controls. Comparison between stable and exacerbated COPD revealed the differential expression of 9 proteins, among which 8 were upregulated and 1 DEP was downregulated (Supplementary Table 4).

**Fig. 2:**
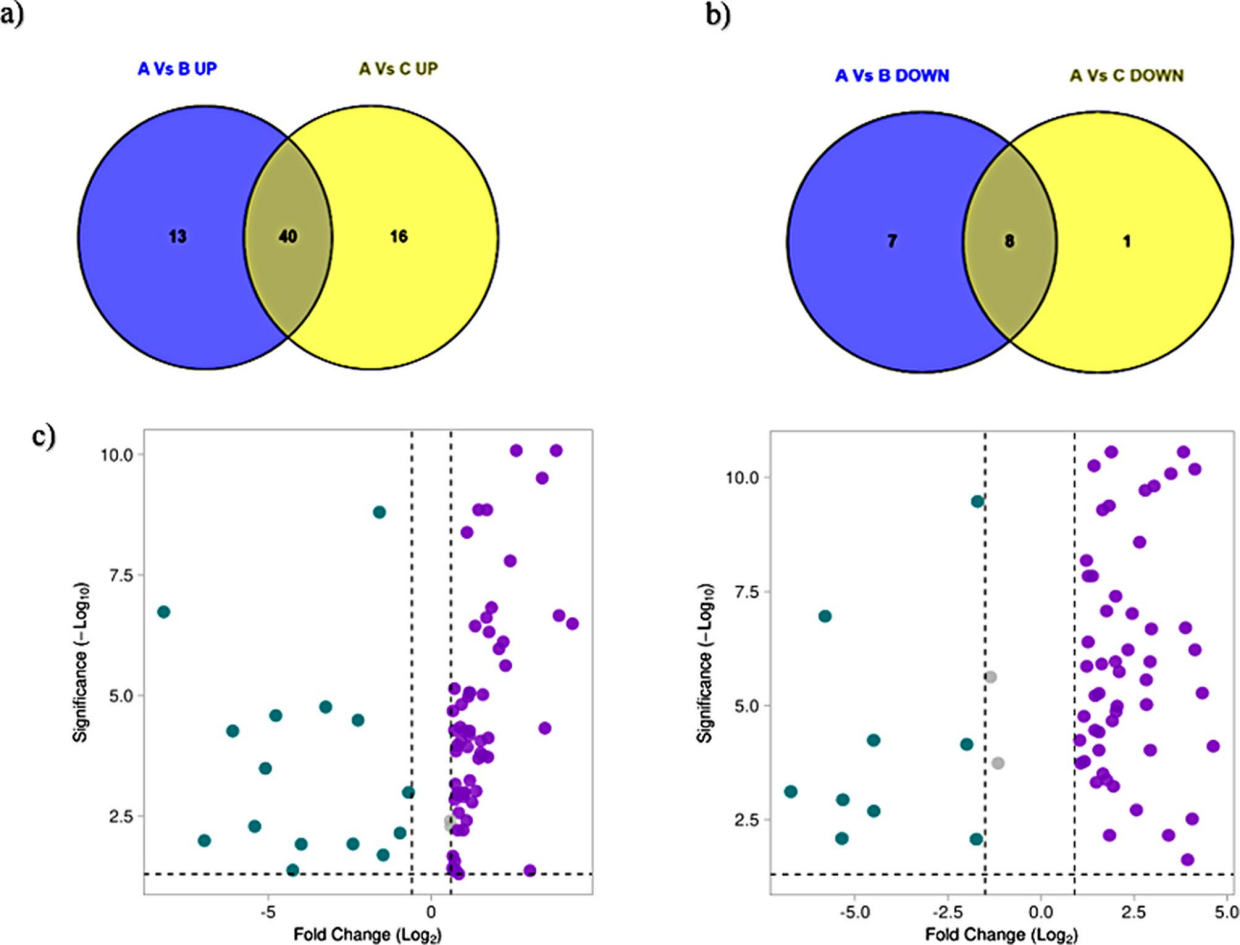
Differential expression of proteins in stable and excaerbated COPD with respect to healthy controls: **(a)** Venn diagram showing the unique and common differentially expressed proteins (DEPs) upregulated in stable and exacerbated COPD with respect to healthy controls. 40 DEPs were commonly up-regulated in stable and excaerbated variants with respect to HC, whereas 13 proteins in stable and 16 proteins in exacerbated COPD were uniquely up-regulated. **(b)** Venn diagram showing the unique and common differentially expressed proteins (DEPs) downregulated in stable and exacerbated COPD with respect to healthy controls. 8 DEPs were commonly downregulated in the COPD variants, whereas 7 proteins in stable and 1 protein in exacerbated COPD were uniquely down-regulated. **(c)** Volcano plot showing the DEPs obtained in stable and exacerbated COPD with respect to control. Upon filtering based on corrected p-value based on FDR, 68 DEPs in stable and 65 proteins in excaerbated COPD showed statistical significance and were taken for further analysis

The Venn results showed 20 unique DEPs in stable COPD and 17 unique DEPs in exacerbated COPD, with 48 DEPs common to both COPD variants with respect to the control (Fig. 2a and b). The stable COPD variant had 53 upregulated and 15 downregulated proteins, whereas the exacerbated COPD variant had 56 upregulated and 9 downregulated proteins, as depicted in the volcano plots shown in Fig. 2c (Supplementary Tables 5 and 6).

**Fig. 3:**
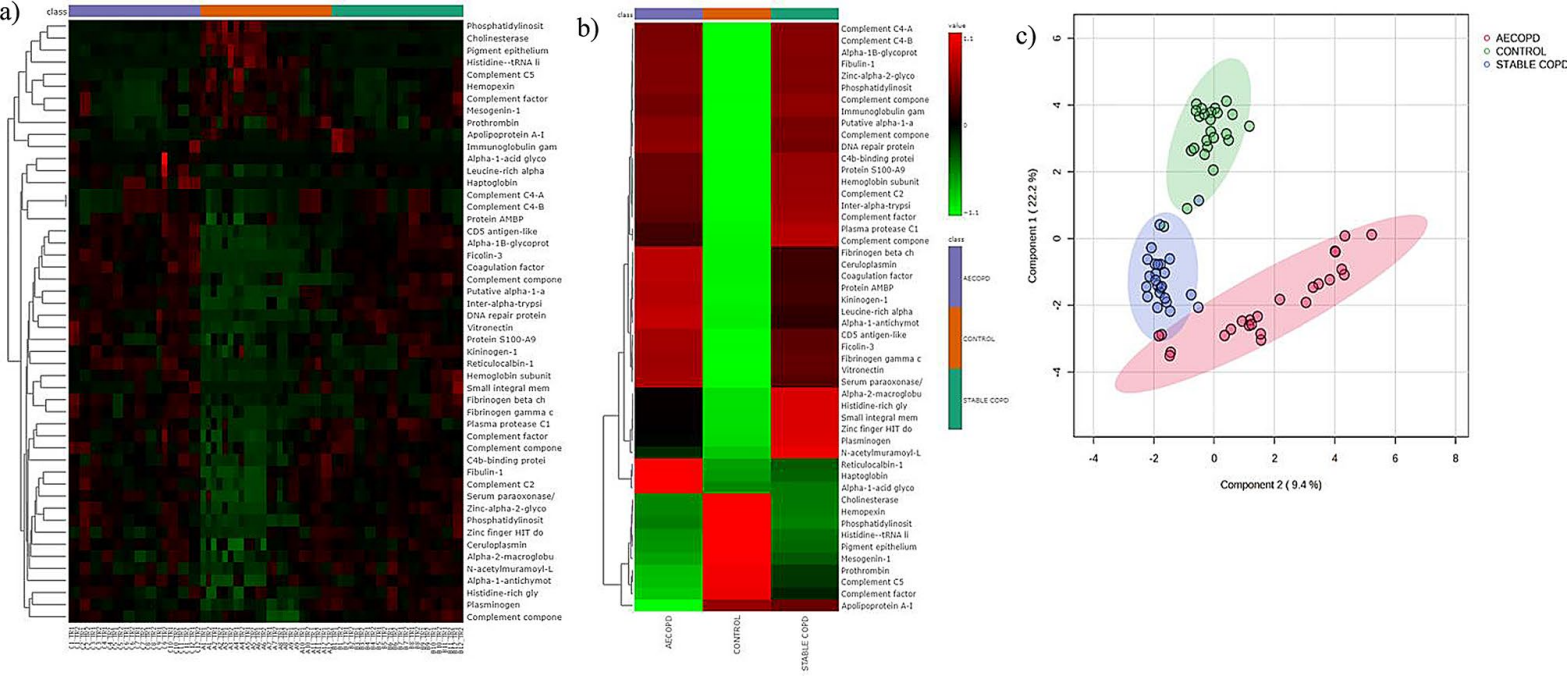
Hierarchical clustering and principal component analysis of the DEPs in stable and exacerbated COPD with respect to healthy controls: **(a)** Heatmap representing the significant DEPs in stable and exacerbated COPD with respect to healthy controls. Clustering shows the co-expression clusters among the differentially expressed proteins. Protein expression levels above the mean levels are in red, protein expression levels below the mean levels are in green. **(b)** Heatmap representing group averages of stable and exacerbated COPD with respect to healthy controls. **(c)** partial least square discriminant analysis (PLS-DA) shows the intra-group homogeneity and the inter-group variability in the three groups. There is clear separation between the exacerbated COPD with the healthy controls

A heatmap constructed to visualize hierarchical clustering depicted the grouping of DEPs according to their expression profiles, as shown in Fig. 3a. Partial least square discriminant analysis (PLS-DA) showed a clear separation between samples of both COPD subgroups with respect to healthy controls, which indicates intergroup variation and intragroup homogeneity, validating the uniformity of samples within groups (Fig. 3b).

**Fig. 4:**
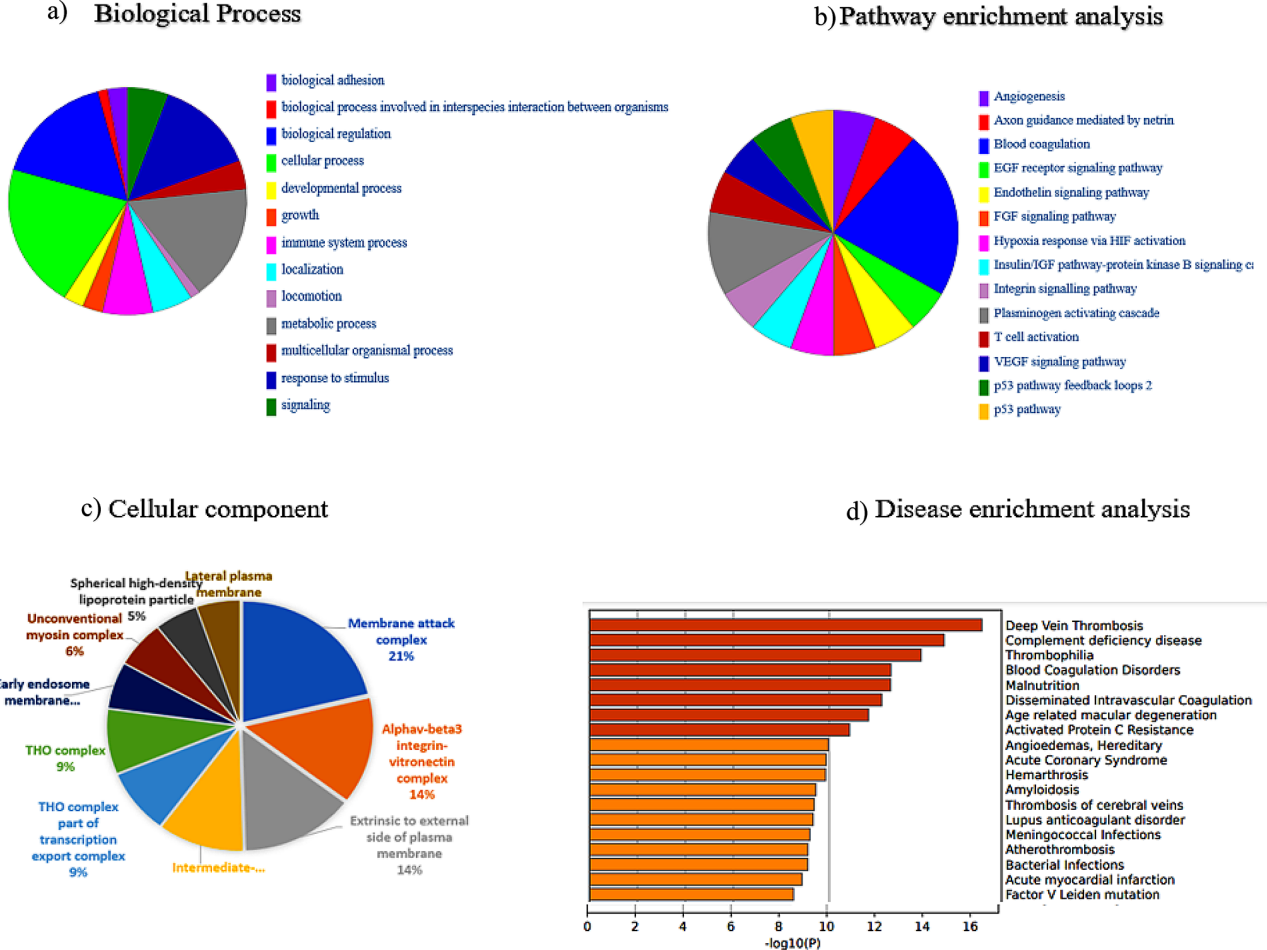
Functional annotation and pathway enrichment analysis of the up-regulated proteins common to stable and exacerbated COPD: **(a)** the major enriched GO terms in biological processes were metabolic process, biological regulation, response to stimulus, cellular process and immune system **(b)** The major overrepresented pathways upon ORA analysis were blood coagulation, plasminogen activating cascade, FGF signalling pathway, endothelin signalling pathway and EGF receptor signalling pathway. **(c)** The DEPs localized to plasma membrane, MAC, THO complex, early endosome and HDL were secreted into plasma. **(d)** Disease over representation analysis showed diseases pertaining to deep vein thrombosis, complement deficiency disease, thrombophilia, blood coagulation disorders, malnutrition and macular degeneration

The major GO BP terms enriched in the upregulated proteins common to stable and exacerbated COPD were metabolic process, biological regulation, response to stimulus, cellular process and immune system process as shown in Fig. 4a.

The most enriched pathways in the Panther overrepresentation analysis (ORA) performed in upregulated proteins across stable and exacerbated groups were blood coagulation, plasminogen activating cascade, FGF signalling pathway, endothelin signalling pathway and EGF receptor signalling pathway (Fig. 4b). Previous studies have reported the role of FGF signalling and endothelin signalling cascades in hypoxia induced pulmonary complications [6]. Disease enrichment analysis revealed the differentially expressed proteins to be overrepresented in deep vein thrombosis, complement deficiency, thrombophilia, blood coagulation disorders, malnutrition, intravascular degeneration and age-related macular degeneration, all complications associated with COPD (Fig. 4d).

GO term enrichment of DEPs in stable and exacerbated COPD was visualized in ClueGO, a Cytoscape plugin; the major functions were complement and coagulation cascades, opsonization, zymogen activation and acute phase response across the two COPD variants (Supplementary figure S3).

Further analysis was performed in the exacerbated COPD group to focus more on the proteins that were differentially expressed in the more severe and life-threatening form of the disease.

**Fig. 5:**
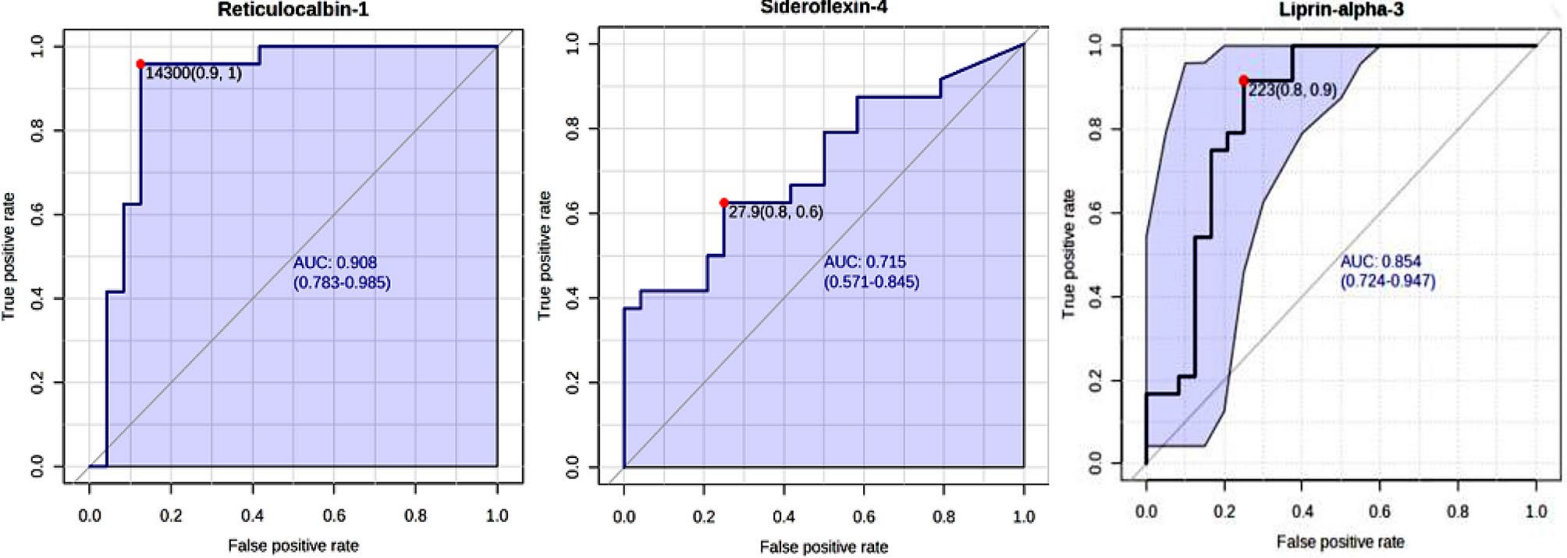
Receiver operator characteristic (ROC) curve of reticulocalbin-1, sideroflexin-4 and liprinα-3 in exacerbated COPD plasma samples: Classical ROC curve for single variable was constructed for the proteins RCN-1, SFXN-4 and LIPα-3 using Metaboanalyst 5.0. RCN-1 showed an area under the curve (AUC) value of 0.908, whereas SFXN-4 showed an AUC value of 0.715 with LIPα-3 showing an AUC value of 0.854. RCN1 and SFXN4 were upregulated in COPD exacerbation whereas LIPα-3 was downregulated in the disease with respect to healthy control, as depicted in the boxplot

Post mass spectrometry, the results were analysed, and two proteins, namely, reticulocalbin-1, and sideroflexin-4 with localized expression in the lungs and bronchus, previously unexplored in COPD, were found to show consistent differential upregulation in all the exacerbated COPD samples with respect to stable COPD and healthy control. Another protein, liprin α-3 showed significant downregulation in COPD with respect to healthy control. The predictive power of these markers was assessed through ROC curve analysis and showed AUC scores of 0.908, 0.715 and 0.856 for reticulocalbin-1, sideroflexin-4 and liprin α-3 respectively (Fig. 5). The area under the curve (AUC) scores in a receiver operator characteristic (ROC) curve is used to analyse the predictive potential of a biomarker candidate. AUC values range between 0 and 1, with higher values indicating better discrimination between the study groups. As a general consensus in analysing a DEP, an AUC score above 0.7 is considered to be a sufficient indication of its predictive capacity, hence validating the biomarker potential of our proteins of interest.

**Fig. 6:**
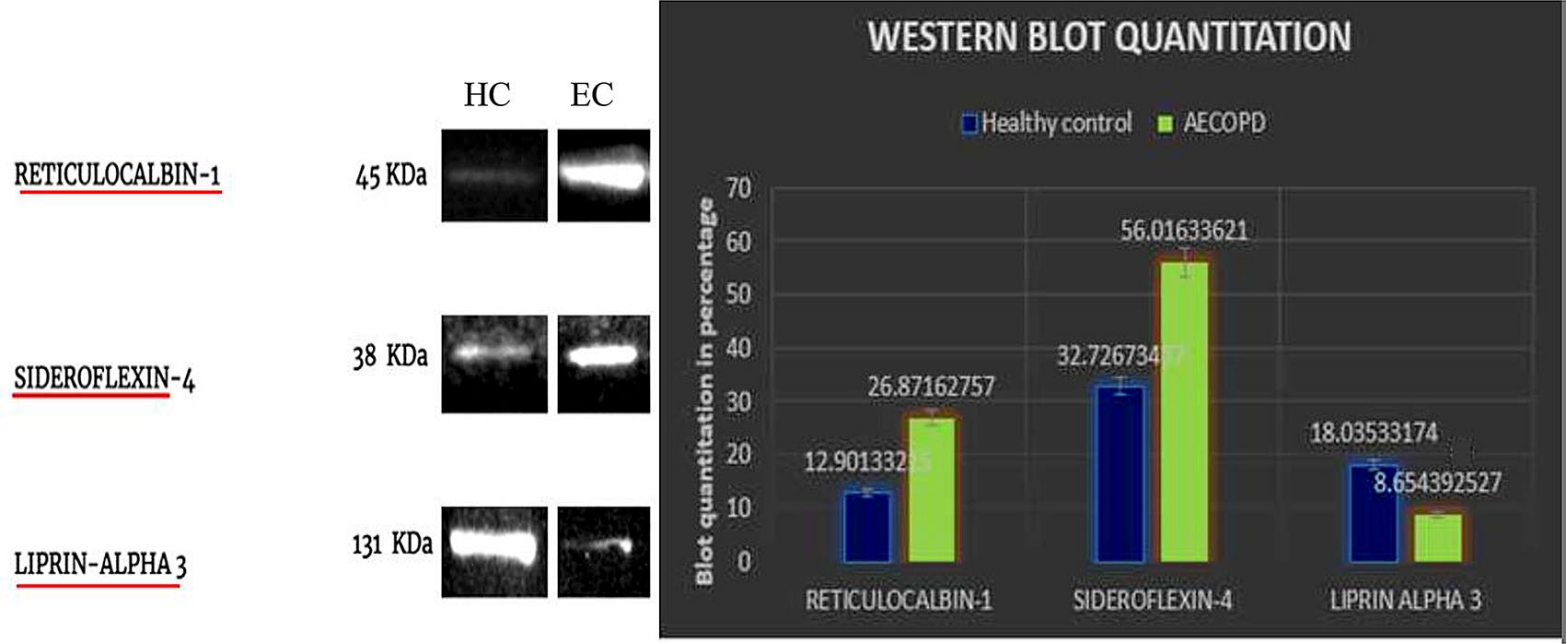
Cross- sectional validation of the screened DEPs using western blot: Western blot analysis of RCN-1, SFXN-4 and LIPα-3 carried out in a validation set (12 HC and 12 AECOPD). The box plot gives the mean of the protein values obtained after blot quantitation expressed in percentage

These proteins were quantitated in a validation cohort using western blotting. Reticulocalbin-1 and sideroflexin-4 showed an increase in band width in exacerbated COPD with respect to healthy controls, whereas liprin α-3 showed a decrease in band width in exacerbated COPD, a confirmation of the mass spectrometric results (Fig. 6). The full blots for the same are given in supplementary figure S4. The up-regulation of unique proteins such as reticulocalbin-1, and sideroflexin-4 could be a contributing factor in the increased severity observed in exacerbated variants of COPD.

## Discussion

COPD is a complex respiratory disorder with high morbidity and mortality rates. Even with the current conventional diagnostic methods, including circulating inflammatory biomarkers, underdiagnosis rates remain high, accounting for a significant gap in the therapeutic management of COPD. The present COPD biomarkers include proteins such as C-reactive protein (CRP), fibrinogen, and RAGE, all of which are indicators of generalized inflammation. Regardless of new research in COPD, a molecular signature/multimarker approach to predict COPD has not seen much progress beyond inflammatory markers such as haptoglobin and fibrinogen. Studies carried out in the recent past have attempted to uncover novel proteins in COPD, but due to a focus on a targeted approach to a single protein biomarker and limitations in the processing of plasma samples, the identification of specific biomarkers and elucidation of COPD pathophysiology has remained inconclusive to date. With 70% underdiagnosis with conventional diagnostic methods, the identification of specific biomarkers that delineate COPD subtypes from healthy controls becomes quintessential.

This study was a label-free plasma proteomics approach that identified differentially expressed protein markers that could delineate exacerbated COPD and stable COPD from healthy controls. The focus of the present study was to use mass spectrometry and bioinformatics to identify future biomarker candidates that are specific to COPD exacerbation and does not indicate generalized inflammation like the existing acute phase reactants projected as COPD biomarkers, namely haptoglobin, serum amyloid protein, RAGE and so on.

### Differential Expression of Generalized Inflammatory Markers induce the production of pro-inflammatory cytokines, leading to persistent inflammation in COPD

The present study observed an upregulation of proteins such as C reactive protein (CRP) in exacerbation COPD and ficolin-3, plasminogen, serum amyloid protein (SAA-P) and complement proteins in both the COPD variants. These proteins are actively involved in acute phase reactions and the production of pro-inflammatory cytokines during inflammation [7]. The study also observed a downregulation of anti-inflammatory proteins such as fetuin-B and pigment epithelium-derived factor in both COPD subtypes [8, 9]. The differential expression of these proteins validated the hyperinflammatory microenvironment in COPD. Nevertheless, all these proteins have been well studied in chronic inflammation and could only serve as generalized markers of inflammation and do not warrant specific diagnosis of COPD exacerbation.

### Elevated expression of adhesion proteins fibulin-1, focadhesin and vitronectin may lead to ECM remodelling in COPD variants

The study observed the upregulation of fibulin-1, focadhesin and vitronectin in both disease variants highlighting the role of adhesion molecules in extracellular matrix remodelling. ECM remodelling affects airway wall thickness and elasticity, leading to bronchoconstriction and breathing difficulties, which are major clinical manifestations in COPD [10, 11].

Analysis of proteomic data revealed the differential expression of nine proteins between stable and exacerbated COPD. The proteins that delineated stable COPD from exacerbation mostly played a role in apoptosis and stress response, inflammatory response, blood coagulation, oxygen transport and hemostasis.

Transiency and reversibility of the stable COPD subgroup prompted us to focus more on the proteins that were differentially expressed in exacerbation COPD delineating it from the stable and healthy control groups.

Reticulocalbin-1, and sideroflexin-4 were the two proteins that showed consistent differential expression and upon further bioinformatics analysis, showed localized expression and noteworthy functionality in COPD exacerbation. Upon ROC analysis, these proteins showed high predictive value in exacerbated COPD with respect to stable COPD and healthy controls.

### Up-regulated RCN-1 could lead to an inhibition of ER stress induced apoptosis leading to persistent inflammation in the lung microenvironment

Reticulocalbin-1 (RCN1) is an endoplasmic reticulum-derived protein known for its role in calcium signalling. It is expressed in macrophages, the respiratory epithelium of the lungs and ciliated cells in the nasopharynx, among other regions. RCN1 showed elevated expression in exacerbation COPD with respect to stable COPD and control samples in the study. Functional annotation combined with a literature review of this interesting protein shed light on its function in inflammation and cell death during ER stress. In exacerbation COPD, in response to an external trigger in the form of pathogens, there is an activation of tumour necrosis factor TNF-α. TNF-α is a pro-inflammatory cytokine that acts as a mediator for the activation of inflammatory signalling pathways such as NF-Kβ and MAPK. A recent study reported that activation of the NF-KB pathway positively correlates with RCN1. Studies report that an overexpressed RCN-1 could lead to inhibition of ER stress-induced apoptosis and inhibition of the unfolded protein response via PERK-IRE-1 signaling, leading to accumulation of unfolded proteins, which would explain persistent inflammation in the cellular microenvironment of the COPD lung. Inhibition of apoptosis and UPR signalling could also lead to the development of autoimmunity, which has not yet been widely studied in COPD. This study also reported the upregulation of MAPK7, further validating the active inflammation in the diseased condition [12].

### Sideroflexin-4 overexpression leading to mitochondrial dysfunction and iron sequestration in airway macrophages could lead to oxidative stress in COPD exacerbation

Sideroflexin-4 (SFXN4) is a mitochondrial protein with moderate expression in respiratory epithelial cells, alveolar cells and macrophages in the lungs and showed consistent elevation in exacerbation COPD in this study. Sideroflexin-4 is a relatively less studied protein, and its functions and interactions are yet to be unmasked. Previous studies in sideroflexin-4 have reported its role in mitochondrial respiration and Fe-S cluster biogenesis. Fe-S clusters are essential cofactors involved in ribosome assembly, DNA repair and electron transport. Excessive Fe-S cluster biogenesis could lead to oxidative stress, mitochondrial dysfunction, DNA damage, iron overload and aggregation of misfolded proteins in the microenvironment, explaining associated clinical manifestations in the COPD lung [13, 14]. An upregulation of sideroflexin-4 would thus explain iron accumulation in the cells and oxidative damage to DNA as iron overload and sequestration by airway macrophages have been previously studied as a major factor in infective COPD exacerbations [15]. The results also showed the upregulation of several DNA repair proteins, such as the DNA repair protein XRCC2, E3 ubiquitin ligase SHPRH and nuclear mitotic apparatus protein 1. This could be explained as a compensatory overexpression of DNA repair proteins in the context of DNA damage in SFXN-4 overexpression.

The differential expression of RCN-1 and SFXN-4 in exacerbation COPD could also implicate the role of COPD pathophysiology in developing a cancer-prone microenvironment in the lungs as these proteins had been previously implicated in the development and progression of many different types of cancers [16].

### The role of Liprin α-3 in muscarinic signalling and airway remodelling in COPD lung

Liprin α-3, another protein previously unexplored in COPD showed consistent downregulation in both stable and exacerbated variants of COPD with respect to control. Liprinα-3 is a scaffold protein showing very low expression in respiratory epithelial cells of the nasopharynx and bronchus. It plays a major role in synapse formation at neuromuscular junctions. Previous reports have shown that a decrease in liprinα-3 leads to a reduction in releasable vesicles at synapses and a reduction in vesicle docking at neuromuscular junctions. A consistent downregulation in liprinα-3 in exacerbation COPD could explain the associated symptoms in COPD related to muscarinic receptor signaling, such as lung secretions, bronchoconstriction and airway remodelling. Liprinα-3 dysregulation could influence acetyl choline-mediated activation of M1 and inhibition of M2 muscarinic receptor signaling, leading to breathing difficulties and mucus hypersecretion. The use of muscarinic antagonists as bronchodilators in COPD treatment in itself is an indication of the role of muscarinic receptors in pulmonary function [17–20]. Liprinα-3, does not have a localized expression in lung tissues and hence does not play a direct role in altering the cellular microenvironment in a COPD lung. However, the consistent differential expression and interesting functionality projects liprinα-3 as a protein of interest that could be explored in the future for its role in COPD pathophysiology.

**Fig. 7:**
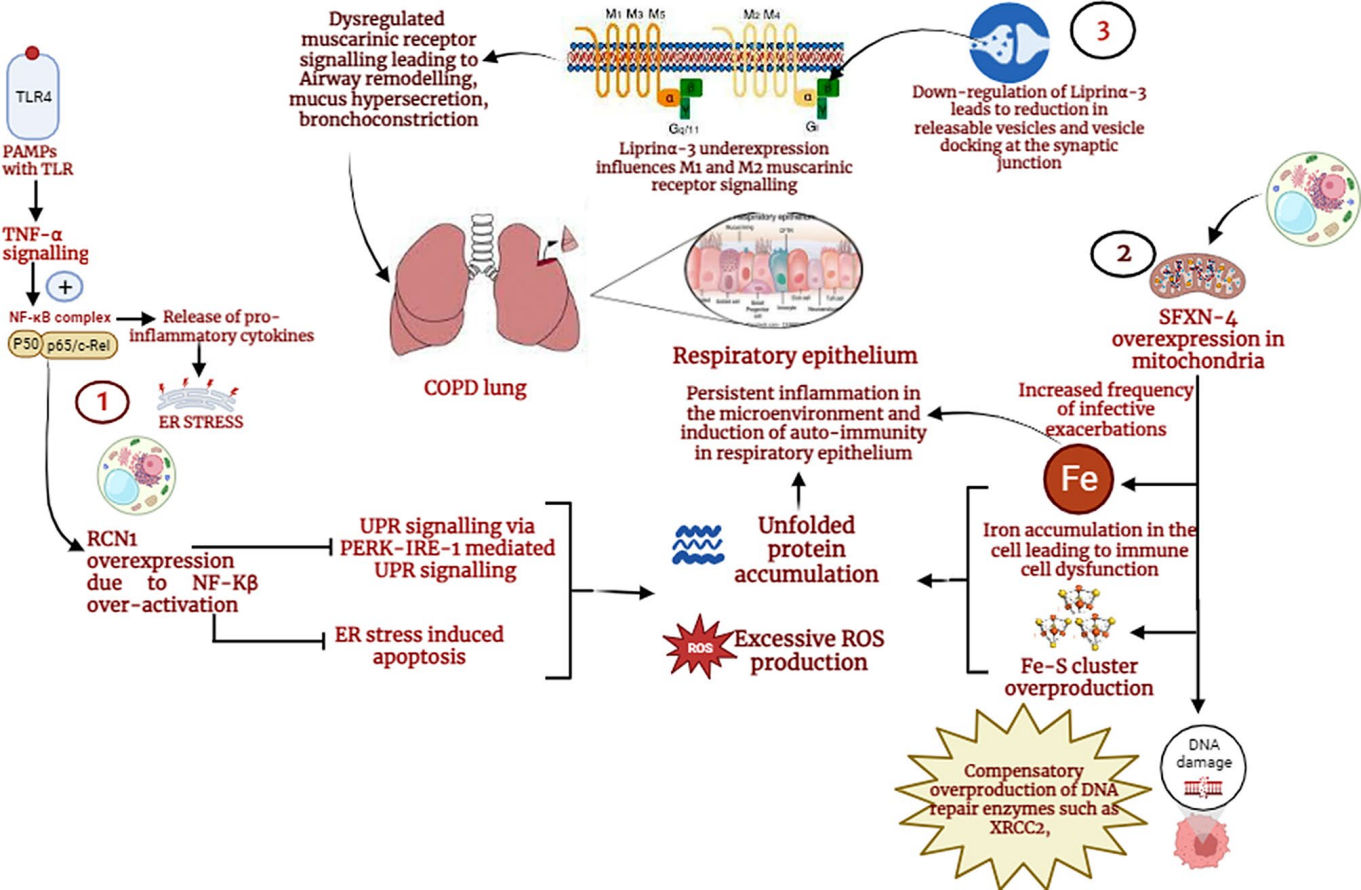
Diagram showing the implication of differential expression of the proteins-RCN-1, SFXN-4 and LIPα-3 in the pathophysiology of exacerbation COPD. Recent literature review and functional annotation of this proteins taken together with their lung specific expression projects them as potential biomarkers for exacerbation COPD

A review of the literature and functional annotation of RCN1, SFXN-4 and LIPα-3 were used to create a hypothetical model of the functional role of these proteins in the COPD lung microenvironment using Biorender (https://www.biorender.com), as presented in Fig. 7.

Our study has focussed on identifying the DEPs in exacerbated COPD in comparison to stable COPD using healthy controls as a baseline reference. In this context, the discovery of the upregulated proteins such as RCN-1 and SFXN-4 holds great promise as candidate biomarkers for exacerbation COPD. The differential expression of the identified proteins is very relevant in the COPD exacerbation pathophysiology as they play important roles in lung function, oxidative stress and persistent inflammation. Future studies using a labelled proteomic approach including more samples from a diverse and widespread population with equal representation from both sexes would contribute greatly to the validation of these proteins as biomarkers of exacerbated Chronic Obstructive Pulmonary Disease.

## Conclusion

The present study discovered a novel protein duo, reticulocalbin-1 and sideroflexin-4, making a breakthrough in understanding the downstream signalling in exacerbation COPD thereby leading to improved clinical management of the disease. Further research into these proteins has the potential to improve clinical monitoring of exacerbation risk, allowing for timely interventions and personalised treatment strategies. The discovery of this previously unexplored protein duo in COPD represents a pioneering work that advances both diagnostic possibilities and opens up new avenues for novel precision therapeutic management of COPD.

### Electronic supplementary material

Below is the link to the electronic supplementary material.

Supplementary Material 1

## Data Availability

The datasets used and/or analysed during the current study and the LC-MS/MS raw files will be made available from the corresponding author on request.

## Abbreviations

COPD: Chronic Obstructive Pulmonary Disease
CAT: COPD Assessment Test
DEP: Differentially expressed proteins
MRC: Medical Research Council dyspnoea scale
GOLD: The Global Initiative for Chronic Obstructive Lung Disease
PLS-DA: Partial Least Square-Discriminant Analysis
PPI: Protein–Protein Interaction
FDR: False Discovery Rate
ROC: Receiver Operator Characteristic Curve
AUC: Area Under the Curve
FEV1/FVC: Forced expiratory volume/Forced vital capacity
