# Supplementary material for: Exploring the novel duo of Reticulocalbin, and Sideroflexin as future biomarker candidates for Exacerbated Chronic Obstructive Pulmonary Disease
Source: Clin Proteomics. 2024 Feb 14;21:10. doi: 10.1186/s12014-024-09459-8 (PMC10865594; doi:10.1186/s12014-024-09459-8)
Supplement: Supplementary file 1 — Supplementary Material 1 [file 12014_2024_9459_MOESM1_ESM.docx]

**SUPPLEMENTARY TABLES AND FIGURES**

b

a

**
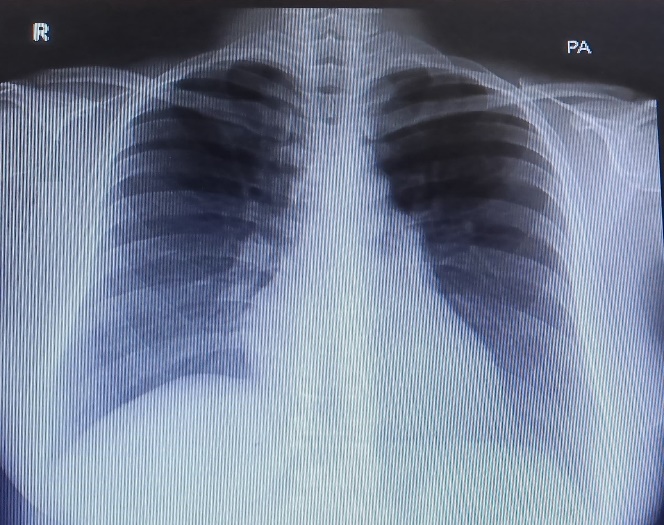

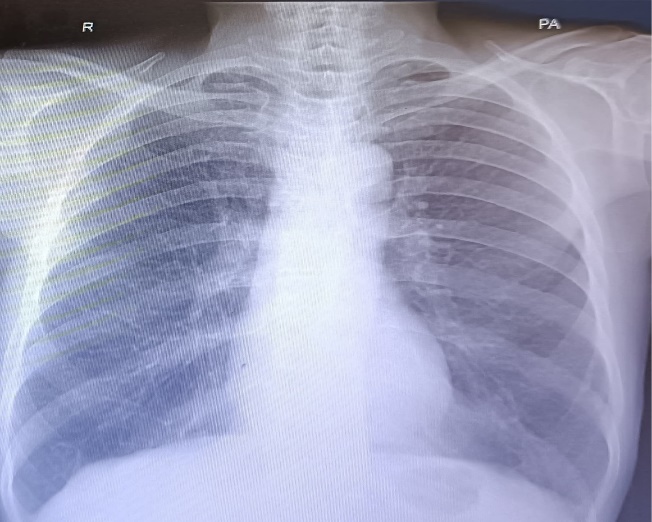
**

**Supplementary figure .1.** Chest X-ray (CXR) of non-COPD patient compared with COPD: a) CXR of a non-COPD. b) CXR of COPD patient


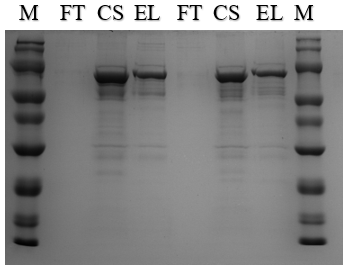


**M- Marker**

**FT-Flowthrough fraction**

**CS-Crude serum**

**EL-Eluted fraction**

**Supplementary figure.2. SDS page of Hu-14 depleted samples:** Flowthrough fractions collected from Hu-14 columns were run through an SDS-PAGE parallel to the eluate and crude serum. The flowthrough fraction shows the removal of all the abundant proteins whereas when in comparison to the eluate and crude serum. This is a validation to the efficiency of the depletion process quintessential to unmask low abundant proteins.

**
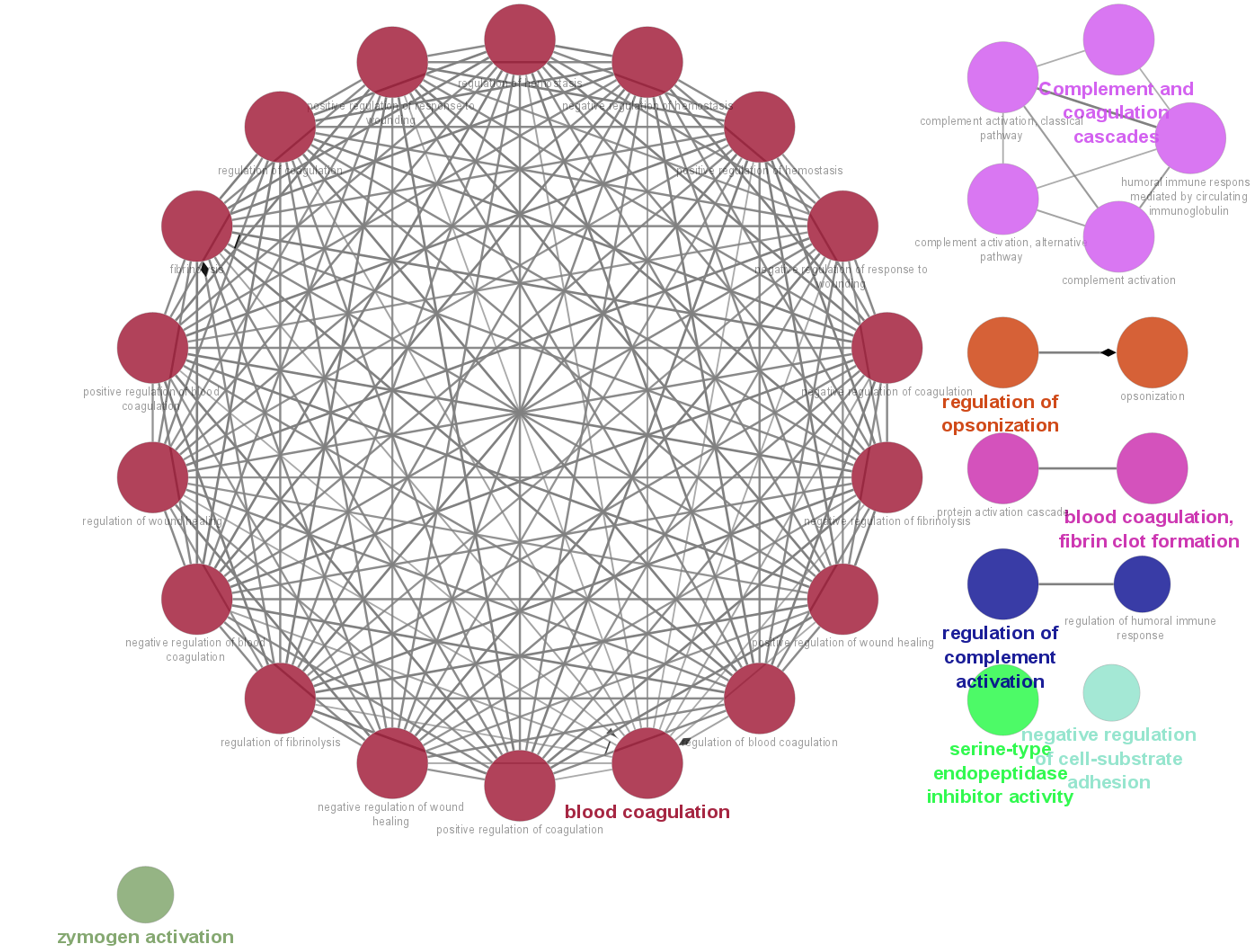

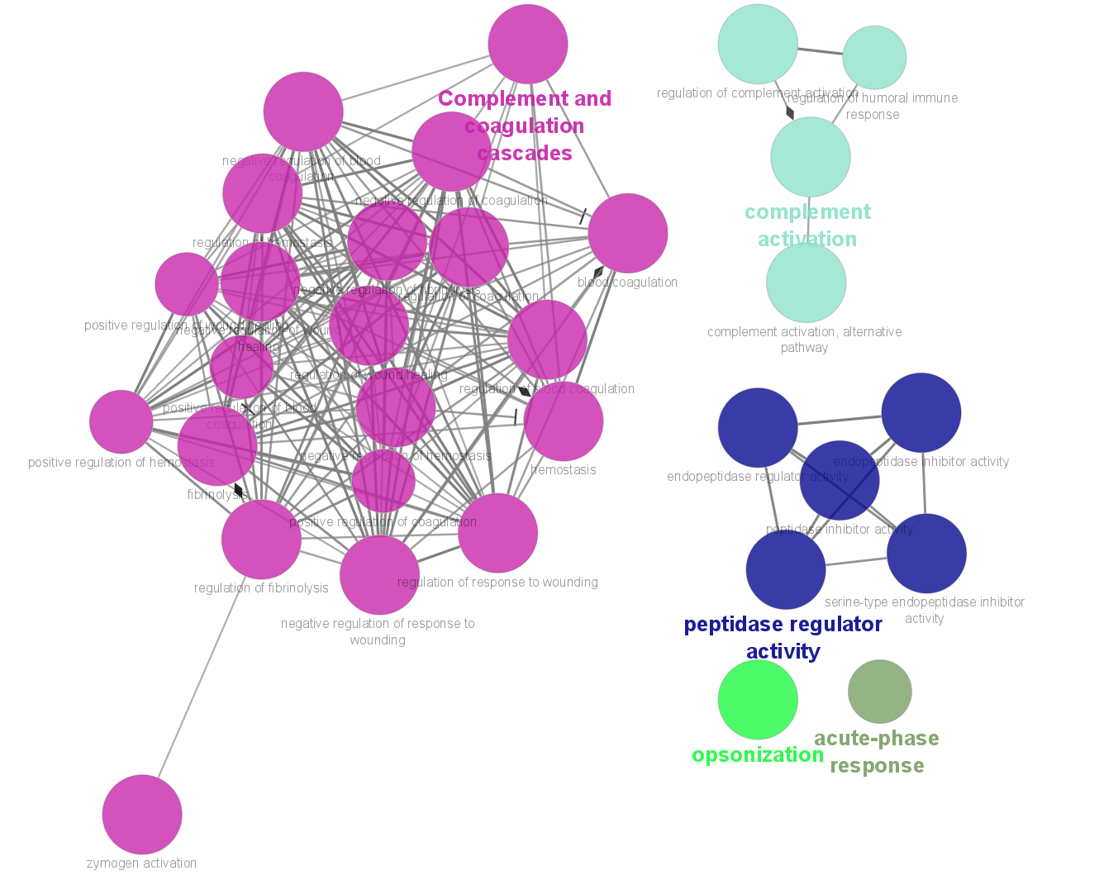
**

b

a

**Supplementary figure.3. ClueGO visulaization of the major biological process enriched in COPD variants:** a) The major BPs enriched in stable COPD are blood coagulation, complement activation, fibrin clot activation, zymogen activation and serine type endopeptidase inhibitor activity. b) Major BPs enriched in exacerbated COPD are peptidase regulator activity, opsonization, acute-phase response, complement activation and coagulation cascades.

a)

**
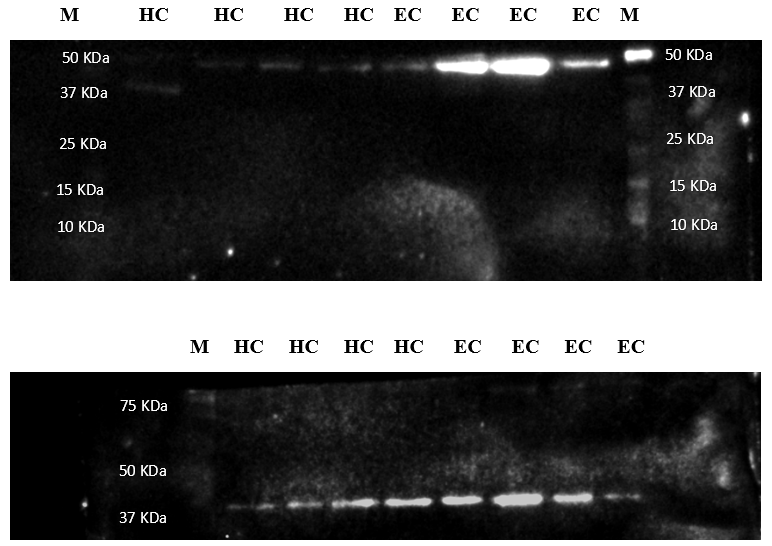
**

c)

b)

**
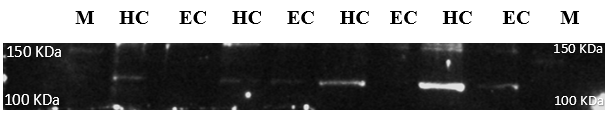
**

**Supplementary figure.4. Original full blots of the representative blot image provided in the main text of a) RCN-1, b) SFXN-4. c) Downregulation of Liprin α-3 samples in exacerbation COPD with respect to healthy controls are also validated through a western blot.**

**Tables**

**Supplementary table.S1. Table showing demographic characteristics of the discovery cohort samples.**

| Variable | Category | HC | SC | EC | p-value |
| --- | --- | --- | --- | --- | --- |
| Gender | Female | 2 | 2 | 2 | - |
|  | Male | 10 | 10 | 10 | - |
| Age | - | 55 ± 0.9428 | 67 ± 5.715 | 65.5± 1.65 | 0.016 |
| CAT score | - | 4.5 ± 1.43 | 12.0 ± 4.4 | 26.4± 7.01 | <0.001 |
| MRC assessment | - | 0 | 1.61± 0.87 | 3.5 ±2.0 | <0.001 |
| ESR |  | 12.33± 2.054 | 24.66± 3.299 | 60.75± 12.67 | 0.001 |
| CRP |  | 0.31 ± 0.014 | 3.0 ± 0.5 | 3.447± 1.940 | 0.042 |
| FEV1/FVC ratio | - | 0.85 ± 0.066 | 0.65± 0.042 | 0.51±0.062 | <0.001 |

CAT-COPD assessment test, MRC -Medical Research Council dyspnoea scale, ESR-erythrocyte sedimentation rate, FEV1/FVC- Forced expiratory volume/Forced vital capacity

**Supplementary table S2: Liquid chromatography gradient with total run time-90 minutes**

| **Time (min)** | **Flow (µl/min)** | **% A (Water)** | **% B (Acetonitrile)** |
| --- | --- | --- | --- |
| Initial | 0.30 | 97.0 | 3.0 |
| 55.50 | 0.30 | 60.0 | 40.0 |
| 63.00 | 0.30 | 20.0 | 80.0 |
| 70.50 | 0.30 | 20.0 | 80.0 |
| 71.10 | 0.30 | 97.0 | 3.0 |
| 90.00 | 0.30 | 97.0 | 3.0 |

**Supplementary table.S3: Table showing demographic characteristics of the validation cohort samples**

| Variable | Category | HC | EC | p-value |
| --- | --- | --- | --- | --- |
| Gender | Female | 2 | 2 | - |
|  | Male | 10 | 10 | - |
| Age | - | 60.5 ± 5.0 | 67.25 ± 4.32 | <0.05 |
| CAT score | - | 4.25 ±1.05 | 23.16 ± 6.8 | <0.001 |
| MRC assessment | - | 0.416 ± 0.07 | 3.37 ± 0.93 | <0.001 |
| ESR | - | 10.58 ± 4.8 | 42.33 ± 20.2 | <0.01 |
| CRP | - | 3.08 ± 1.6 | 17.10 ± 9.25 | <0.001 |
| FEV1/FVC ratio | - | 0.92 ± 0.05 | 0.53 ± 0.07 | <0.001 |

CAT-COPD assessment test, MRC -Medical Research Council dyspnoea scale, ESR-erythrocyte sedimentation rate,

FEV1/FVC- Forced expiratory volume/Forced vital capacity

**Supplementary table S4: List of differentially expressed proteins in exacerbated COPD with respect to stable COPD ( C Vs B)**

| Sl no | Accession | Peptide count | Unique peptides | q Value | Max fold change | Description |
| --- | --- | --- | --- | --- | --- | --- |
| Up-regulated proteins | | | | | | |
| 1 | Q8NGE5 | 2 | 1 | 1.98E-13 | 5463.424274 | Olfactory receptor 10A7 |
| 2 | O14733 | 2 | 1 | 1.84E-09 | 8.091002671 | Dual specificity mitogen-activated protein kinase kinase 7 |
| 3 | P00915 | 4 | 2 | 2.79E-09 | 7.341233748 | Carbonic anhydrase 1 |
| 4 | P08571 | 2 | 2 | 0.00011 | 5.182943092 | Monocyte differentiation antigen CD14 |
| 5 | O00159 | 2 | 1 | 0.00373 | 2.219124486 | Unconventional myosin-Ic |
| 6 | P15169 | 14 | 12 | 7.41E-05 | 2.201419238 | Carboxypeptidase N catalytic chain |
| 7 | P69905 | 7 | 6 | 0.00016 | 2.073069374 | Hemoglobin subunit alpha |
| 8 | P03951 | 9 | 5 | 0.00046 | 1.64684013 | Coagulation factor XI |
| Down-regulated proteins | | | | | | |
| 9 | P05160 | 18 | 11 | 0.00176 | 2.048282592 | Coagulation factor XIII B chain |

**Supplementary table S5: List of differentially expressed proteins in stable COPD with respect to control ( B Vs A)**

| **Sl no** | **Accession** | **Peptide count** | **Unique peptides** | **q Value** | **Max fold change** | **Description** |
| --- | --- | --- | --- | --- | --- | --- |
| **Up-regulated proteins** | | | | | | |
| 1 | O75636 | 5 | 3 | 1.33E-12 | 5.340948004 | Ficolin-3 |
| 2 | Q5VW36 | 3 | 1 | 2.52E-09 | 20.01305306 | Focadhesin |
| 3 | P20742 | 13 | 1 | 2.52E-09 | 15.06312997 | Pregnancy zone protein |
| 4 | Q96PQ7 | 5 | 2 | 6.32E-11 | 14.2046963 | Kelch-like protein 5 |
| 5 | Q8N3J2 | 2 | 1 | 8.58E-06 | 11.16864133 | N(6)-adenine-specific methyltransferase METTL4 |
| 6 | O43543 | 3 | 2 | 6.32E-11 | 10.5458117 | DNA repair protein XRCC2 |
| 7 | P00740 | 6 | 4 | 1.83E-07 | 6.075715758 | Coagulation factor IX |
| 8 | A2RUS2 | 4 | 1 | 3.63E-06 | 4.832086761 | DENN domain-containing protein 3 |
| 9 | P61769 | 5 | 2 | 3.89E-07 | 4.605269442 | Beta-2-microglobulin |
| 10 | P00488 | 5 | 4 | 6.05E-06 | 3.343790476 | Coagulation factor XIII A chain |
| 11 | O43866 | 12 | 10 | 1.69E-08 | 3.243713056 | CD5 antigen-like |
| 12 | P20848 | 4 | 1 | 1.43E-06 | 2.856623195 | Putative alpha-1-antitrypsin-related protein |
| 13 | Q149N8 | 7 | 3 | 9.78E-05 | 2.840992303 | E3 ubiquitin-protein ligase SHPRH |
| 14 | P04217 | 52 | 45 | 8.00E-10 | 2.728026372 | Alpha-1B-glycoprotein |
| 15 | O00750 | 6 | 2 | 2.26E-06 | 2.7252949 | Phosphatidylinositol 4-phosphate 3-kinase C2 domain-containing subunit beta |
| 16 | P03951 | 12 | 3 | 0.00019 | 2.589943853 | Kinesin-like protein KIF21B |
| 17 | P10643 | 51 | 42 | 2.09E-08 | 2.543934048 | Complement component C7 |
| 18 | A0A096LP01 | 3 | 1 | 0.000338 | 2.373968382 | Small integral membrane protein 26 |
| 19 | P07358 | 23 | 18 | 4.51E-06 | 2.262233637 | Complement component C8 beta chain |
| 20 | P25311 | 46 | 35 | 5.81E-05 | 2.252457993 | Zinc-alpha-2-glycoprotein |
| 21 | Q9BYX2 | 22 | 13 | 2.96E-05 | 2.241224092 | TBC1 domain family member 2A |
| 22 | P19823 | 70 | 57 | 4.62E-07 | 2.232289035 | Inter-alpha-trypsin inhibitor heavy chain H2 |
| 23 | P00747 | 77 | 67 | 7.78E-06 | 2.194814639 | Plasminogen |
| 24 | Q96PD5 | 27 | 25 | 8.58E-06 | 2.157292729 | N-acetylmuramoyl-L-alanine amidase |
| 25 | P04004 | 39 | 32 | 4.54E-08 | 2.130838462 | Vitronectin |
| 26 | P22792 | 20 | 16 | 0.001548 | 2.10720021 | Carboxypeptidase N subunit 2 |
| 27 | Q14980 | 21 | 9 | 1.48E-05 | 2.024922645 | Nuclear mitotic apparatus protein 1 |
| 28 | Q13188 | 5 | 2 | 6.30E-05 | 2.022628256 | Serine/threonine-protein kinase 3 |
| 29 | P02743 | 17 | 15 | 6.63E-05 | 1.967532958 | Serum amyloid P-component |
| 30 | P05155 | 34 | 24 | 0.00019 | 1.967512412 | Plasma protease C1 inhibitor |
| 31 | P08185 | 17 | 12 | 0.015674 | 1.885425749 | Corticosteroid-binding globulin |
| 32 | P42702 | 7 | 4 | 1.48E-05 | 1.84619872 | Leukemia inhibitory factor receptor |
| 33 | P01344 | 4 | 2 | 0.007319 | 1.78962459 | Insulin-like growth factor II |
| 34 | O75882 | 44 | 23 | 0.000168 | 1.788154129 | Attractin |
| 35 | P05090 | 9 | 6 | 9.78E-05 | 1.767854734 | Apolipoprotein D |
| 36 | Q9UHR6 | 12 | 10 | 0.000211 | 1.753759174 | Zinc finger HIT domain-containing protein 2 |
| 37 | Q8IWJ2 | 19 | 6 | 0.00019 | 1.731728483 | GRIP and coiled-coil domain-containing protein 2 |
| 38 | P00742 | 18 | 12 | 9.78E-05 | 1.68635428 | Coagulation factor X |
| 39 | Q6P4A7 | 3 | 1 | 0.014395 | 1.669010371 | Sideroflexin-4 |
| 40 | P07357 | 23 | 19 | 0.000915 | 1.663371565 | Complement component C8 alpha chain |
| 41 | Q06033 | 30 | 25 | 0.000581 | 1.646732159 | Inter-alpha-trypsin inhibitor heavy chain H3 |
| 42 | P05160 | 18 | 11 | 0.005433 | 1.643851369 | Coagulation factor XIII B chain |
| 43 | P00751 | 103 | 78 | 1.62E-05 | 1.6336168 | Complement factor B |
| 44 | P02750 | 23 | 19 | 0.000311 | 1.63302565 | Leucine-rich alpha-2-glycoprotein |
| 45 | P02656 | 2 | 2 | 0.000454 | 1.607892697 | Apolipoprotein C-III |
| 46 | P23142 | 14 | 13 | 0.000545 | 1.604680424 | Fibulin-1 |
| 47 | Q96IY4 | 5 | 3 | 0.000946 | 1.583901348 | Carboxypeptidase B2 |
| 48 | P27918 | 11 | 8 | 0.000729 | 1.580411836 | Properdin |
| 49 | P04003 | 33 | 27 | 0.00019 | 1.578392472 | C4b-binding protein alpha chain |
| 50 | P06681 | 47 | 33 | 0.000512 | 1.567465675 | Complement C2 |
| 51 | P14151 | 2 | 1 | 0.039945 | 1.517945359 | L-selectin |
| 52 | P04196 | 67 | 61 | 0.003032 | 1.511870191 | Histidine-rich glycoprotein |
| 53 | P02748 | 36 | 26 | 0.000471 | 1.502073449 | Complement component C9 |
| **Down-regulated proteins** | | | | | | |
| 54 | Q8NGE5 | 2 | 1 | 1.33E-12 | 835.4690883 | Olfactory receptor 10A7 |
| 55 | P0C860 | 3 | 2 | 1.21E-05 | 68.24460061 | Putative male-specific lethal-3 protein-like 2 |
| 56 | Q5K130 | 2 | 1 | 5.11E-06 | 33.96013172 | Putative uncharacterized protein CLLU1-AS1 |
| 57 | O75145 | 4 | 1 | 8.18E-06 | 2.725387143 | Liprin-alpha-3 |
| 58 | Q9Y5F1 | 6 | 2 | 0.002367 | 15.87025332 | Protocadherin beta-12 |
| 59 | P49590 | 2 | 1 | 2.78E-07 | 9.442856941 | Histidine--tRNA ligase_ mitochondrial |
| 60 | P80108 | 13 | 7 | 0.004417 | 4.719011071 | Phosphatidylinositol-glycan-specific phospholipase D |
| 61 | P41219 | 4 | 2 | 0.030785 | 3.969475221 | Peripherin |
| 62 | P06276 | 6 | 4 | 2.52E-09 | 3.009966113 | Cholinesterase |
| 63 | P22352 | 7 | 6 | 0.014853 | 2.781735536 | Glutathione peroxidase 3 |
| 64 | Q4G0S7 | 3 | 2 | 0.028974 | 2.637366442 | Coiled-coil domain-containing protein 152 |
| 65 | P49321 | 4 | 2 | 0.048335 | 2.493109512 | Nuclear autoantigenic sperm protein |
| 66 | P36955 | 25 | 18 | 0.00019 | 1.946704088 | Pigment epithelium-derived factor |
| 67 | Q9UGM5 | 4 | 3 | 0.038349 | 1.722130599 | Fetuin-B |
| 68 | P02775 | 3 | 1 | 0.002301 | 1.621517348 | Platelet basic protein |

**Supplementary table S6: List of differentially expressed proteins in exacerbated COPD with respect to control ( C Vs A)**

| **Sl no** | **Accession** | **Peptide count** | **Unique peptides** | **Corrected p-value** | **Max fold change** | **Description** |
| --- | --- | --- | --- | --- | --- | --- |
| **Up-regulated proteins** | | | | | | |
| 1 | O75636 | 5 | 3 | 5.90E-13 | 6.278618968 | Ficolin-3 |
| 2 | Q5VW36 | 3 | 1 | 3.13E-07 | 24.72589869 | Focadhesin |
| 3 | Q8N3J2 | 2 | 1 | 2.39E-08 | 17.61566088 | N(6)-adenine-specific methyltransferase METTL4 |
| 4 | Q96PQ7 | 5 | 2 | 3.13E-11 | 17.58055789 | Kelch-like protein 5 |
| 5 | Q6P4A7 | 3 | 1 | 0.00350453 | 15.34119205 | Sideroflexin-4 |
| 6 | Q5K130 | 2 | 1 | 2.23E-08 | 14.7516997 | Putative uncharacterized protein CLLU1-AS1 |
| 7 | P20742 | 13 | 1 | 1.56E-11 | 14.20860337 | Pregnancy zone protein |
| 8 | O43543 | 3 | 2 | 7.97E-12 | 11.23632957 | DNA repair protein XRCC2 |
| 9 | P00740 | 6 | 4 | 2.78E-08 | 8.213177461 | Coagulation factor IX |
| 10 | P00488 | 5 | 4 | 2.42E-07 | 7.1247925 | Coagulation factor XIII A chain |
| 11 | P00915 | 4 | 2 | 1.91E-09 | 7.10259932 | Carbonic anhydrase 1 |
| 12 | O14733 | 2 | 1 | 2.42E-07 | 5.470919028 | Dual specificity mitogen-activated protein kinase kinase 7 |
| 13 | A2RUS2 | 4 | 1 | 2.12E-06 | 5.049289 | DENN domain-containing protein 3 |
| 14 | O75037 | 12 | 3 | 2.80E-06 | 4.286521845 | Kinesin-like protein KIF21B |
| 15 | P61769 | 5 | 2 | 1.12E-06 | 4.128941359 | Beta-2-microglobulin |
| 16 | Q149N8 | 7 | 3 | 1.77E-06 | 4.006321171 | E3 ubiquitin-protein ligase SHPRH |
| 17 | O43866 | 12 | 10 | 6.61E-10 | 3.707747923 | CD5 antigen-like |
| 18 | P02741 | 6 | 4 | 0.046095147 | 3.614534201 | C-reactive protein |
| 19 | Q0JRZ9 | 5 | 4 | 2.47E-08 | 3.549089244 | F-BAR domain only protein 2 |
| 20 | P22792 | 20 | 16 | 0.000174304 | 3.394940385 | Carboxypeptidase N subunit 2 |
| 21 | Q13188 | 5 | 2 | 1.49E-07 | 3.388025967 | Serine/threonine-protein kinase 3 |
| 22 | P07358 | 23 | 18 | 4.47E-10 | 3.150366692 | Complement component C8 beta chain |
| 23 | Q15293 | 4 | 2 | 9.75E-06 | 3.097931749 | Reticulocalbin-1 |
| 24 | O75882 | 44 | 23 | 1.92E-07 | 2.944197725 | Attractin |
| 25 | Q96IY4 | 5 | 3 | 9.17E-05 | 2.94210981 | Carboxypeptidase B2 |
| 26 | P20848 | 4 | 1 | 4.99E-07 | 2.941115614 | Putative alpha-1-antitrypsin-related protein |
| 27 | O00750 | 6 | 2 | 1.77E-06 | 2.730119158 | Phosphatidylinositol 4-phosphate 3-kinase C2 domain-containing subunit beta |
| 28 | P04217 | 52 | 45 | 1.35E-10 | 2.688065362 | Alpha-1B-glycoprotein |
| 29 | P10643 | 51 | 42 | 4.54E-10 | 2.597385885 | Complement component C7 |
| 30 | P04004 | 39 | 32 | 2.49E-08 | 2.329203715 | Vitronectin |
| 31 | P25311 | 46 | 35 | 5.53E-05 | 2.243754324 | Zinc-alpha-2-glycoprotein |
| 32 | Q9BYX2 | 22 | 13 | 1.51E-05 | 2.225924058 | TBC1 domain family member 2A |
| 33 | P08571 | 2 | 2 | 0.000142129 | 2.096254405 | Monocyte differentiation antigen CD14 |
| 34 | P19823 | 70 | 57 | 6.28E-07 | 2.058715191 | Inter-alpha-trypsin inhibitor heavy chain H2 |
| 35 | Q6I9Y2 | 3 | 2 | 0.000123083 | 1.989347954 | THO complex subunit 7 |
| 36 | P02750 | 23 | 19 | 0.000166149 | 1.944445928 | Leucine-rich alpha-2-glycoprotein |
| 37 | Q8IWJ2 | 19 | 6 | 1.56E-05 | 1.925806947 | GRIP and coiled-coil domain-containing protein 2 |
| 38 | P08185 | 17 | 12 | 0.001725431 | 1.893722061 | Corticosteroid-binding globulin |
| 39 | O00159 | 2 | 1 | 0.001750423 | 1.86769564 | Unconventional myosin-Ic |
| 40 | P00742 | 18 | 12 | 1.56E-05 | 1.853798549 | Coagulation factor X |
| 41 | P02743 | 17 | 15 | 9.80E-05 | 1.782728622 | Serum amyloid P-component |
| 42 | P05155 | 34 | 24 | 9.42E-05 | 1.710951465 | Plasma protease C1 inhibitor |
| 43 | A0A096LP01 | 3 | 1 | 0.00036002 | 1.70977301 | Small integral membrane protein 26 |
| 44 | Q14980 | 21 | 9 | 0.001007566 | 1.669191649 | Nuclear mitotic apparatus protein 1 |
| 45 | Q15165 | 2 | 1 | 0.000265413 | 1.628555622 | Serum paraoxonase/arylesterase 2 |
| 46 | P29622 | 19 | 11 | 0.001791138 | 1.625479062 | Kallistatin |
| 47 | P01042 | 58 | 43 | 9.29E-05 | 1.622787205 | Kininogen-1 |
| 48 | P23142 | 14 | 13 | 0.000476462 | 1.602717484 | Fibulin-1 |
| 49 | P03951 | 9 | 5 | 0.001313218 | 1.586163916 | Coagulation factor XI |
| 50 | P00747 | 77 | 67 | 0.000549143 | 1.577118222 | Plasminogen |
| 51 | P07225 | 25 | 17 | 0.012950389 | 1.576524919 | Vitamin K-dependent protein S |
| 52 | P02656 | 2 | 2 | 0.000465229 | 1.562148533 | Apolipoprotein C-III |
| 53 | P01011 | 71 | 59 | 0.000764007 | 1.549792569 | Alpha-1-antichymotrypsin |
| 54 | P61626 | 7 | 3 | 0.000921532 | 1.54559621 | Lysozyme C |
| 55 | P00751 | 103 | 78 | 0.000221416 | 1.525067452 | Complement factor B |
| 56 | P04003 | 33 | 27 | 0.000549143 | 1.51488661 | C4b-binding protein alpha chain |
| **Down-regulated proteins** | | | | | | |
| 57 | P49590 | 2 | 1 | 6.93E-11 | 55.75248186 | Histidine--tRNA ligase_ mitochondrial |
| 58 | Q9Y5F1 | 6 | 2 | 0.000154795 | 40.28627981 | Protocadherin beta-12 |
| 59 | O75145 | 4 | 1 | 8.89E-06 | 22.59897094 | Liprin-alpha-3 |
| 60 | P0C860 | 3 | 2 | 0.000221416 | 22.54346263 | Putative male-specific lethal-3 protein-like 2 |
| 61 | P80108 | 13 | 7 | 0.005071205 | 4.473798981 | Phosphatidylinositol-glycan-specific phospholipase D |
| 62 | P06276 | 6 | 4 | 4.47E-10 | 3.252868178 | Cholinesterase |
| 63 | Q9UGM5 | 4 | 3 | 0.003338763 | 2.464837132 | Fetuin-B |
| 64 | P36955 | 25 | 18 | 7.53E-06 | 2.223276574 | Pigment epithelium-derived factor |
| 65 | Q6ZN30 | 17 | 10 | 0.017694875 | 1.647154294 | Zinc finger protein basonuclin-2 |
